# Immune cell profiles of metastatic HER2-positive breast cancer patients according to the sites of metastasis

**DOI:** 10.1007/s10549-021-06447-6

**Published:** 2021-11-24

**Authors:** Tiia J. Honkanen, Milla E. K. Luukkainen, Antti Tikkanen, Peeter Karihtala, Markus Mäkinen, Juha P. Väyrynen, Jussi P. Koivunen

**Affiliations:** 1 Department of Oncology and Radiotherapy, Oulu University Hospital, POB 20, 90029 Oulu, Finland; 2 Department of Pathology, Oulu University Hospital, POB 21, 90029 Oulu, Finland; 3 Medical Research Center Oulu, POB 5000, 90014 Oulu, Finland; 4 Cancer and Translational Medicine Research Unit, University of Oulu, POB 5000, 90014 Oulu, Finland; 5 Department of Oncology, University of Helsinki and Helsinki University Comprehensive Cancer Center, Helsinki, Finland

**Keywords:** Breast cancer, HER2, Liver metastasis, Tumour profiles

## Abstract

**Purpose:**

Recent works have characterized that metastatic site can affect the tumour immune profiles and efficiency of cancer immunotherapies. The prognosis of HER2-positive breast cancer is associated with the characteristics of the tumour immune microenvironment, with immunological cells playing a central role in efficiency of HER2-targeted antibodies. Here we investigated the prognostic significance of different metastatic sites and their correlation to tumour immune profiles in HER2-positive breast cancer treated with trastuzumab.

**Methods:**

We collected all (*n* = 54) HER2-positive metastatic breast cancer patients treated with trastuzumab containing regimens at Oulu University Hospital 2009–2014. Pathological and clinical data were collected from electronic patient records. The tumour immune profiles were analysed from pre-treatment primary tumours using well-characterized immunological markers with computer-assisted immune cell counting.

**Results:**

Of the metastatic sites, only liver metastases were associated with poor prognosis (hazard ratio 1.809, 95% confidence interval 1.004–3.262), especially when presented as the primary site of metastases. Of the other sites, pulmonary metastases characterized a patient profile with trend to improved survival. Of the studied tumour immunological markers, patients with liver metastases had low densities of CD3^+^ T cells (*p* = 0.030) and M1-like macrophages in their primary tumours (*p* = 0.025). Of the other studied markers and sites, patients with pulmonary metastases had low STAB1^+^-immunosuppressive macrophage density in their primary tumours.

**Conclusion:**

Our results suggest that the site of metastasis is associated with prognosis in HER2-positive breast cancer, highlighted by the poor prognosis of liver metastases. Furthermore, liver metastases were associated with adverse tumour immune cell profiles.

**Supplementary Information:**

The online version contains supplementary material available at 10.1007/s10549-021-06447-6.

## Introduction

Around 20% of all breast cancers have amplification of the human epidermal growth factor receptor 2 (*HER2*), which had been associated with aggressive tumour type and poor prognosis, before targeted therapies for HER2 were developed [1–4]. Immunology is suggested to play a major role in influencing the prognosis in HER2-positive breast cancer and treatment efficiency of HER2-targeted antibodies. The mode of action of HER2 antibodies is partly related to antibody-dependent cell cytotoxicity (ADCC) in which T cells mediate the target cell killing [5, 6].

The appearance of distant metastases is associated with higher mortality in breast cancer [7]. In all subtypes of breast cancer, the most common sites of metastasis are bone, liver, lung, and brain. Bone metastases have often been linked to better prognosis, whereas patients with brain metastases are known to have the worst prognosis, especially in de novo stage IV breast cancers [8, 9]. The metastatic pattern varies in different breast cancer subtypes; all subtypes are prone to metastasize to the bone, but brain and liver metastases are more often seen in HER2 positive and triple-negative breast cancers [8–12].

A recent study by Yu et al. [13] showed that liver metastasis can affect systemic anti-tumoural immunity by recruiting immunosuppressive macrophages into liver, which leads to deletion of cytotoxic T cells and diminishes the efficacy of immune checkpoint inhibitors. Our previous studies with metastatic HER2-positive breast cancer have demonstrated a strong association of cytotoxic T cells and M1-like macrophages in primary tumours with better prognosis [14, 15]. Since immunology has a central role in HER2-positive breast cancer and targeted treatment acts through immune-mediated mechanisms [5], we speculate that the presence of liver metastases would accordingly be associated with adverse prognosis and specific tumour immune profiles of the primary tumour.

In the current study, we tested this hypothesis by evaluating the prognosis and tumour immune cell profiles of 54 patients with metastatic HER2-positive breast cancer treated with trastuzumab containing regimens.

## Results

### Patients and Samples

We searched pharmacy records of Oulu University Hospital and identified 54 patients who had received intravenous trastuzumab for metastatic breast cancer at least once during 2009–2014. Median age of the patients at the time of diagnosis was 58 years and 37 (68.5%) of the patients had oestrogen receptor-positive tumours (Table 1). Median overall survival and median survival in metastatic disease were 58 and 39 months, respectively. Of all the patients 22 (40.7%) had primary metastatic disease at the time of diagnosis and 32 (59.3%) had relapsed disease.

**Table 1:** Patient demographics (*n* = 54)

	*n* (%)
Oestrogen receptor positivity	37 (68.5)
Primary metastatic disease	22 (40.7)
*Liver metastasis*	20 (37.0)
Primary liver metastasis	12 (22.2)
*Pulmonary metastasis*	20 (37.0)
Primary pulmonary metastasis	17 (31.5)
*Brain metastasis*	19 (35.2)
Primary brain metastasis	2 (3.7)
*Bone metastasis*	29 (53.7)
Primary bone metastasis	25 (46.3)
Median age at diagnosis, years (range)	58 (28–82)
Median survival, months (range)	58 (4–242)
Median survival in metastatic disease, months (range)	39 (0–217)

The most common site of metastasis was bone, which was observed in 29 patients (53.7%) and 25 of whom (46.3%) had bone as a primary site of metastasis. Liver and pulmonary metastases were both seen in 20 patients (37.0%). Of the patients with liver metastasis, 12 (22.2%) had liver as the primary site of metastasis, whereas 17 (31.5%) of the patients with pulmonary metastasis had lungs as the primary site of metastasis. Brain metastasis was observed in 19 patients (35.2%), but only 2 (3.7%) had brain as the primary site of metastasis (Table 1).

In our previous studies with metastatic HER2^+^ breast cancer[14, 15], we found that high infiltration of both CD8^+^ T cells and M1-like macrophages in the centre of the tumour (CT) were associated with improved survival. We updated the follow-up time and status of the patients and reanalysed the survival estimates. High density of CD8^+^ T cells in CT (HR 0.321, 95% CI 0.163—0.634), high density of M1-like macrophages in CT (HR 0.339, 95% CI 0.161—0.715), and the combination of CD8^+^ T cells and M1-like macrophages in CT (high density of CD8 or M1 in CT; HR 0.246, 95% CI 0.094—0.641) and high density of CD8 and M1 in CT (HR 0.082, 95% CI 0.025—0.272) predicted improved survival. With this updated follow-up, the survival differences were more pronounced than in our previous studies (Supplementary figure is provided in the Online Resource 1).

### Prognosis of the patients according to the metastatic sites

**Fig. 1:**
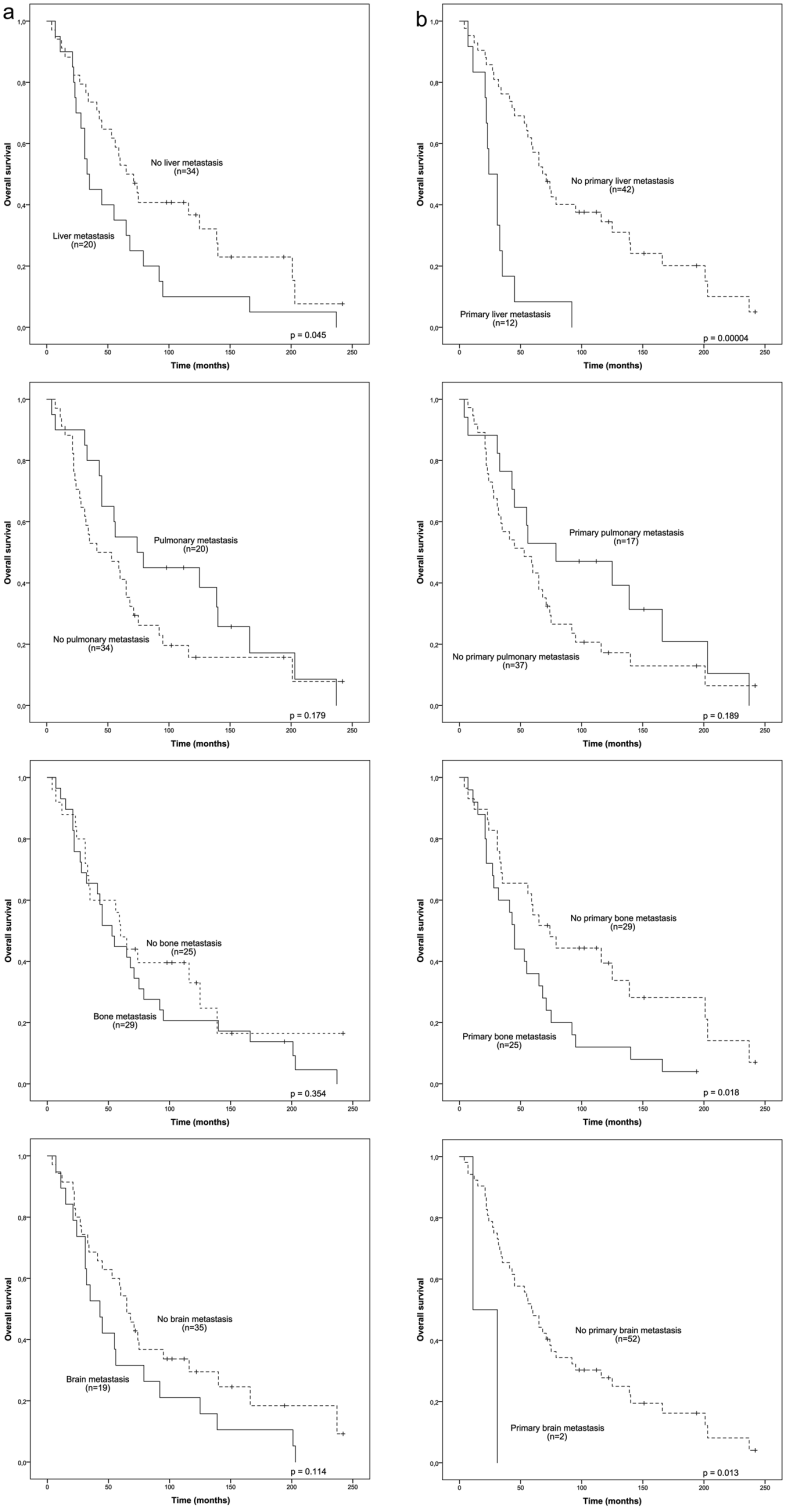
Survival analysis of metastatic HER2^+^ breast cancer patients according to the sites of metastasis. **A** Kaplan–Meier estimates demonstrate the difference in the overall survival of metastatic HER2^+^ breast cancer patients with or without liver, pulmonary, bone, and brain metastases. **B** Kaplan–Meier estimates illustrate the overall survival according to the first site of metastasis. Crosses mark censored events

Bone, liver, lungs, and brain were the most common sites of metastasis in this study and these were selected for further analysis. The impact of the metastatic sites to the overall survival of the patients during the whole disease course were investigated with Kaplan–Meier analysis. Liver, bone, and brain metastases were linked with worse prognosis; however, only liver metastasis were statistically significant (HR 1.809, 95% CI 1.004 – 3.262, Fig. 1a). Interestingly, pulmonary metastasis showed tendency towards improved survival (HR 0.662, 95% CI 0.361 – 1.216) but this, however, was not statistically significant (Fig. 1a).

Next, we evaluated whether the primary site of metastasis would also be linked to survival differences. We observed that liver (HR 4.171, 95% CI 2.009 – 8.656), bone (HR 2.047, 95% CI 1.115 – 3.757), and brain (HR 5.317, 95% CI 1.202 – 23.528) as a primary site of metastases were linked to poor prognosis, whereas primary pulmonary metastasis again showed tendency towards improved survival (HR 0.655, 95% CI 0.346 – 1.239) (Fig. 1b). We also carried out a multivariate analysis with liver as primary metastasis site and adjuvant trastuzumab treatment, the strongest prognostic factor in the cohort. The adverse prognosis of primary liver metastasis retained (5.131, 95% CI 1.505 – 17.493) in multivariate analysis (Table 2).

**Table 2:** Univariate and multivariate analyses for survival

	Univariate		Multivariate	
	HR	95% CI	HR	95% CI
*Adjuvant trastuzumab*				
Yes vs no	6.268	2.331–16.852	5.633	2.034–15.597
*Liver as primary site of metastasis*				
Yes vs no	4.171	2.009–8.656	5.131	1.505–17.493

### Correlation of the immune profiles and metastasis sites

Next, we wanted to question, whether different metastatic sites were associated with variable primary tumour immune profiles. Since the survival of the patients with liver and pulmonary metastases varied most considerably, we chose these two sites for further analysis on immune profiles. Even though the sites of primary metastasis showed more significant survival differences, the number of primary cases was too low for reliable statistical analysis and we therefore chose to analyse patients with either liver or pulmonary metastasis during the whole follow-up. We included numerous immunology-related cell types (markers) in the analysis: T cells (CD3), cytotoxic T cells (CD8), regulatory T cells (FoxP3), NK cells (CD56), M1-like (iNOS) and M2-like (CD163) macrophages, immunosuppressive STAB1^+^ macrophages, and IDO1^+^ immune cells. We also evaluated IDO1 and CD47 expression in tumour cells. The scoring of the immunological cells was carried out using computer-assisted counting of the positively stained cells/mm^2^, whilst tumour cell markers were scored according to staining intensity or counted histoscore (based on the proportion of positive tumour cells and staining intensity).

**Fig. 2:**
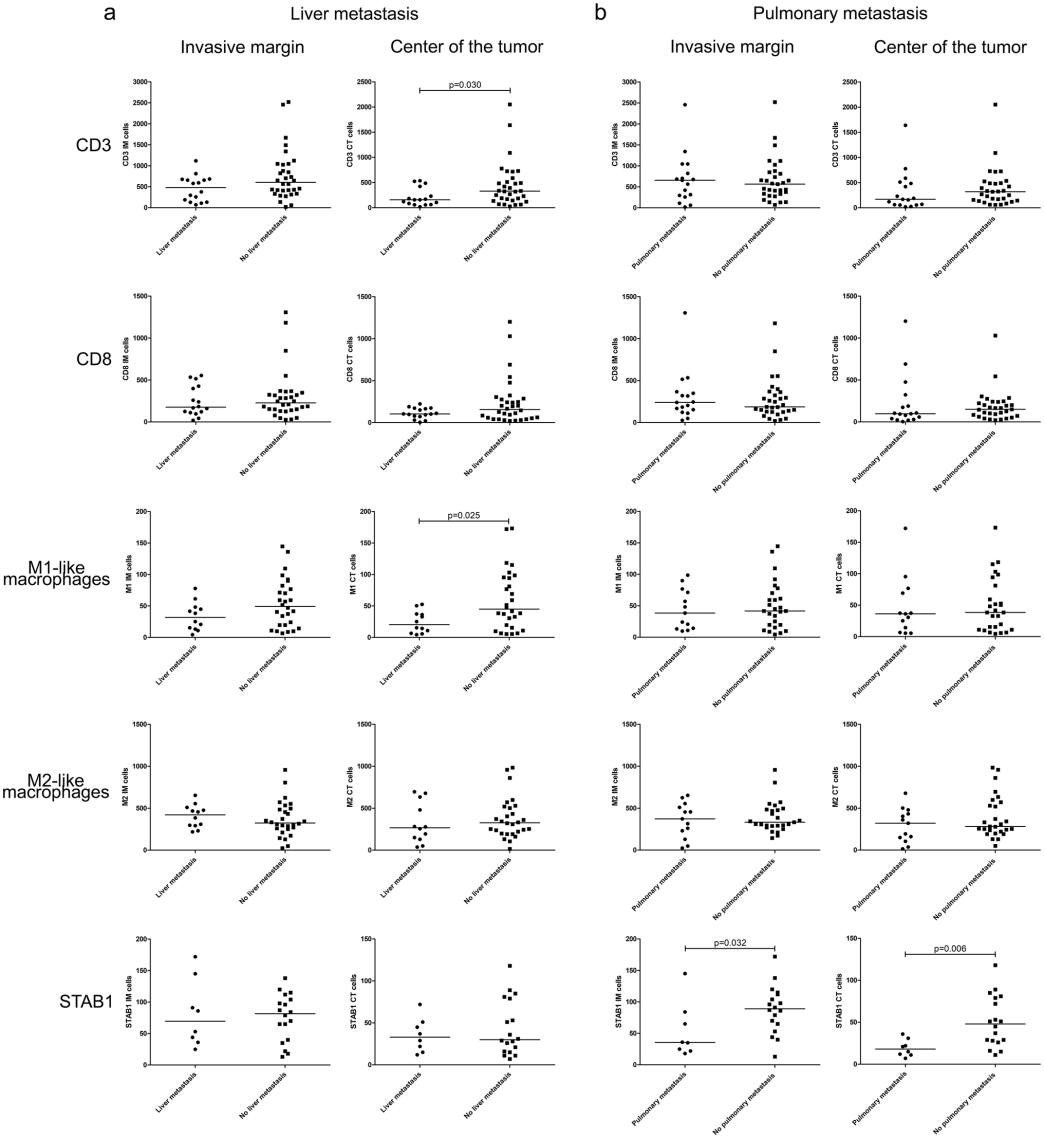
Immune cell densities in the invasive margin and centre of the tumour in metastatic HER2^+^ patients with liver or pulmonary metastasis. Quantity of CD3, CD8, M1-like macrophages, M2-like macrophages, and STAB1^+^ cells in the invasive margin and centre of the tumour according to liver metastasis **A** or pulmonary metastasis **B** status. Median values for each immune cell types are marked as vertical lines. Mann–Whitney test was used to compare the median values of two groups; only significant *p*-values are presented

T cells and M2-like macrophages were the most abundant cell types both in the invasive margin (IM) and centre of the tumour (CT) (Table 3). Mann–Whitney test was used to compare the median immune cell densities of the tumours, both in the IM and CT, in patient with or without liver, and with or without pulmonary metastasis. Patients with liver metastasis had lower amount of CD3^+^ T cells in the CT (*p* = 0.030) and M1-like macrophages in the CT (*p* = 0.025) than patients without liver metastasis (Fig. 2a). In addition, patients with pulmonary metastasis had lower amount of STAB1^+^ macrophages in the IM (*p* = 0.032) and CT (*p* = 0.006) (Fig. 2b). The other studied immunological markers did not show any statistically significant associations with liver or pulmonary metastasis (data not shown).

**Table 3:** Median densities of the studied immune cells

	All cases, *n* = 54 (range) (cells/mm^2^)	Liver metastasis, *n* = 20 (range) (cells/mm^2^)	Pulmonary metastasis, *n* = 20 (range) (cells/mm^2^)
CD3 IM	573 (20 – 2520)	479 (71 – 1120)	659 (20 – 2459)
CD3 CT	242 (19 – 2051)	158 (19 – 537)	170 (19 – 1641)
CD8 IM	207 (20 – 1307)	176 (20 – 554)	240 (27 – 1307)
CD8 CT	120 (4 – 1201)	103 (4 – 223)	97 (4 – 1201)
iNOS IM	42 (4 – 145)	32 (4 – 78)	38 (10 – 99)
iNOS CT	37 (4 – 173)	20 (4 – 53)	36 (6 – 172)
CD163 IM	338 (24 – 957)	421 (219 – 653)	372 (24 – 653)
CD163 CT	295 (15 – 983)	267 (37 – 695)	321 (15 – 678)
STAB1 IM	82 (13 – 172)	70 (25 – 172)	36 (18 – 145)
STAB1 CT	30 (7 – 118)	33 (12 – 72)	18 (7 – 36)

## Discussion

Previous works have suggested that different anatomical locations of metastasis may correlate with tumour immunology. Especially liver metastasis has been linked to poor immunological tumour profiles and responses to immune checkpoint inhibitor treatments [13, 16]. Tumour immunology has a central role in the prognosis of HER2-positive breast cancer and the cornerstone of its treatment is the HER2-targeted antibodies which effect significantly through immune activation [6, 17]. Therefore, we questioned whether the HER2-positive breast cancer with different metastatic sites would have altered survival and tumour immune profiles. We have previously shown that especially high number of tumor CD8-positive T cells and M1-like macrophages are associated with good prognosis in this disease [14, 15].

The association of different metastatic sites to survival has been extensively studied in breast cancers. Brain metastases have generally been associated with the poorest prognosis, whilst bone metastases have been linked to better prognosis [8, 9, 18–20]. The association between metastatic sites and prognosis has less frequently been studied in HER2-positive breast cancer but there is some evidence suggesting similar survival tendencies as in other breast cancers [8]. Results of the current study suggest that liver metastases are associated with the poorest prognosis, whilst patients with pulmonary metastases showed the best prognosis. The adverse survival association was the most significant, when patient presented with liver as the primary site of metastasis. Since our study only included patients who were treated with trastuzumab, the study might not capture patients with the poorest prognosis such as extensive brain metastasis who were never treated with systemic therapy. Our findings may suggest that the efficacy of HER2 antibody treatment might differ according to the metastatic site. Since liver metastasis is thought to induce general immunosuppression [13, 16] and HER2 antibodies mediate their effects through antibody-dependent cellular cytotoxicity (ADCC) [6, 17], immunosuppression might be accounting for the observed results. Importantly, however, our study includes quite recent patients and the treatment paradigms of HER2-positive breast cancer have been evolving in the last years with introduction of new HER2 antibodies and antibody-linked cytotoxic payloads and older studies might not capture the effects of these treatments.

Tumour immune cell profiles have been widely studied in recent years. Especially high density of cytotoxic T cells has been associated with good prognosis in numerous cancers [21–23]. We have previously characterized the immune cell profiles in HER2-positive breast cancer and have shown that especially, high densities of cytotoxic T cells and M1-like macrophages in the centre of the tumour region are strong independent prognostic markers surpassing all the studied baseline factors for survival [14, 15]. Here, we studied a large number of immunological markers in primary tumours and investigated their correlation to metastatic sites. As we hypothesized, we were able to detect differences in the immune microenvironment of the primary tumours according to the metastatic site. In brief, liver metastasis was associated with low densities of T cells and M1-like macrophages in the centre of the tumour, suggestive for an immunological desert or excluded-type tumour microenvironment. Furthermore, pulmonary metastasis was associated with low STAB1^+^ macrophage density. STAB1 is an immunosuppressive marker associated with M2-like macrophages. One could speculate that altered tumour immunological microenvironment could account for the survival differences of the metastatic sites.

Our study has some weaknesses and strengths. The patient cohort was retrospectively collected, was limited in size, and adequate primary tumour samples were not available for all the analyses. The low number of cases did not enable us to study tumour immune profiles amongst the primary metastasis profiles which were the most prominently associated with prognosis. Therefore, the results should be verified in a larger, preferably prospectively collected, patient cohort. Another field of interest would be to study the pairwise tumour immune profiles from neo-adjuvant or relapse setting and verify whether the profiles alter in response to the treatment or time. However, the pairwise samples were available only in few patients and we could not investigate this further. In the analysis for specific markers, we used only single, however, well-characterized antibodies. We used computer-assisted cell counting which has been shown to surpass manual counting methods [24] and we analysed the markers in different tumour locations. Our patient cohort is extensively characterized and has a long follow-up and excellent outcomes with median survival of 58 months.

We envision that our study results could have potential clinical value. The study suggests that patient’s prognosis in metastatic HER2-positive breast cancer can be defined by analysing the metastasis profiles. Furthermore, liver metastases define a patient group with poor prognosis and these patients also bare poor immunological features of the primary tumour. One could speculate that if the treatment results with the existing HER2-targeted therapies are ineffective with general immunosuppressive features of liver metastasis, better treatment outcomes could potentially be achieved with combinatory approaches or novel agents. Our results suggest that patients can be stratified according to the tumour immune profile and novel treatment should be investigated especially in adjuvant or neo-adjuvant settings of patients with poor prognosis features. Interestingly, previous works have characterized the role of M2-like macrophages in the liver metastasis-associated immunosuppression [13] and some M2-like targeting agents are under clinical evaluation [25]. Interestingly, our study showed an association between low densities of STAB1-positive cells, a marker for immunosuppressive macrophages, with good prognosis-associated pulmonary metastasis. STAB1 targeting is also currently investigated in an early-phase clinical cancer trial (NCT03733990).

In conclusion, the current study investigated prognosis of HER2-positive breast cancer according to metastasis sites and their associations with primary tumour immune profiles. We showed that liver metastasis is associated with poor prognosis and adverse tumour immune profiles. These results suggest that metastatic sites might characterize immunologically variable patient subgroups and these could be used to stratify patients when investigating new treatment options.

## Materials and methods

### Patient data

The patients for this retrospective study were identified through pharmacy records of Oulu University Hospital as previously described [26]. In short, all the patients who had received intravenous trastuzumab for metastatic breast cancer and had adequate pre-chemotherapy samples available were selected for the study (*n* = 54). HER2 positivity was determined by the presence of *HER2* amplification in chromogenic in situ hybridization.

Most of the clinical data were gathered previously [14, 15, 26] but were updated for this study including the metastatic sites and the survival status in 01/2021. Overall survival was calculated from the time of diagnosis to death or end of follow-up. Survival in metastatic disease was calculated from the time of radiological or histological identification of metastatic disease to death or end of follow-up. Primary site of metastasis was defined as the site of metastasis at the time of diagnosis of metastatic disease.

### Immunohistochemistry and immune cell counting

Immunohistochemical analyses of CD3, CD8, FoxP3, CD56, iNOS, CD163, IDO1, and CD47 have been described in previous studies [14, 15]. CD3, CD8, and CD56 staining were performed for 48 samples, FoxP3, iNOS, CD163, IDO1, and CD47 for 40 samples, and STAB1 for 26 samples due to a restriction of the tumour material. Immunohistochemical analysis of STAB1 was conducted on 3.5 µm sections cut from paraffin-embedded specimens. The sections were first deparaffinized in HistoClear and rehydrated through graded alcohols. Antigen retrieval was performed with proteinase K (Agilent DAKO, #S3020) for 5 min. Endogenous peroxidase activity was neutralized in 1% H_2_0_2_ aqua solution for 20 min after which the samples were blocked using Vectastain ABC-HRP rat kit (Vector Laboratories, #PK-6140). Primary antibody anti-STAB1 was a kind gift from Maija Hollmén (MediCity, University of Turku) and the samples were incubated with the antibody overnight + 4 °C. Bound antibodies were detected using the Vectastain ABC-HRP rat kit. Diaminobenzidine (DAB) was used as the chromogen and hematoxylin as the counterstain.

For the analysis of the immunohistochemistry, the tissue sections were scanned with Aperio AT2 image-capturing device (Leica Biosystems). Imagescope (Aperio Technologies) software, version 11.2 was used to view the scanned images and capture 3–6 (median 6) images from the centre of the tumour (CT) and the invasive margin (IM) for immune cell counting. The criteria for the different tumour locations are previously described [14]. The cells were counted as earlier described and validated [24] by computer-assisted counting method, which utilizes ImageJ software.

### Statistics

IBM SPSS Statistics 24.0 for Windows (IBM Corporation, Armonk, NY, USA) was used for statistical analyses. Survival analyses were performed with Kaplan–Meier method using log-rank test. In univariate and multivariate analyses, Cox regression was used and results were reported with 95% confidence level. Continuous variables between two groups were compared with Mann–Whitney test and 2-tailed *p*-values were used. GraphPad Prism version 5 (GraphPad Software, San Diego, CA, USA) was used to create vertical scatter plots with median lines.

## Supplementary Information

Below is the link to the electronic supplementary material.

Supplementary file1 (PDF 85 KB)

## Data Availability

All data generated or analysed during this study are included in this published article. The raw data used in the analyses of the current study are not publicly available due to legislative issues concerning the privacy of the patients.
