# Supplementary material for: Immune cell profiles of metastatic HER2-positive breast cancer patients according to the sites of metastasis
Source: Breast Cancer Res Treat. 2021 Nov 24;191(2):443–50. doi: 10.1007/s10549-021-06447-6 (PMC8763933; doi:10.1007/s10549-021-06447-6)
Supplement: Supplementary file 1 — Supplementary file1 (PDF 85 KB) [file 10549_2021_6447_MOESM1_ESM.pdf]

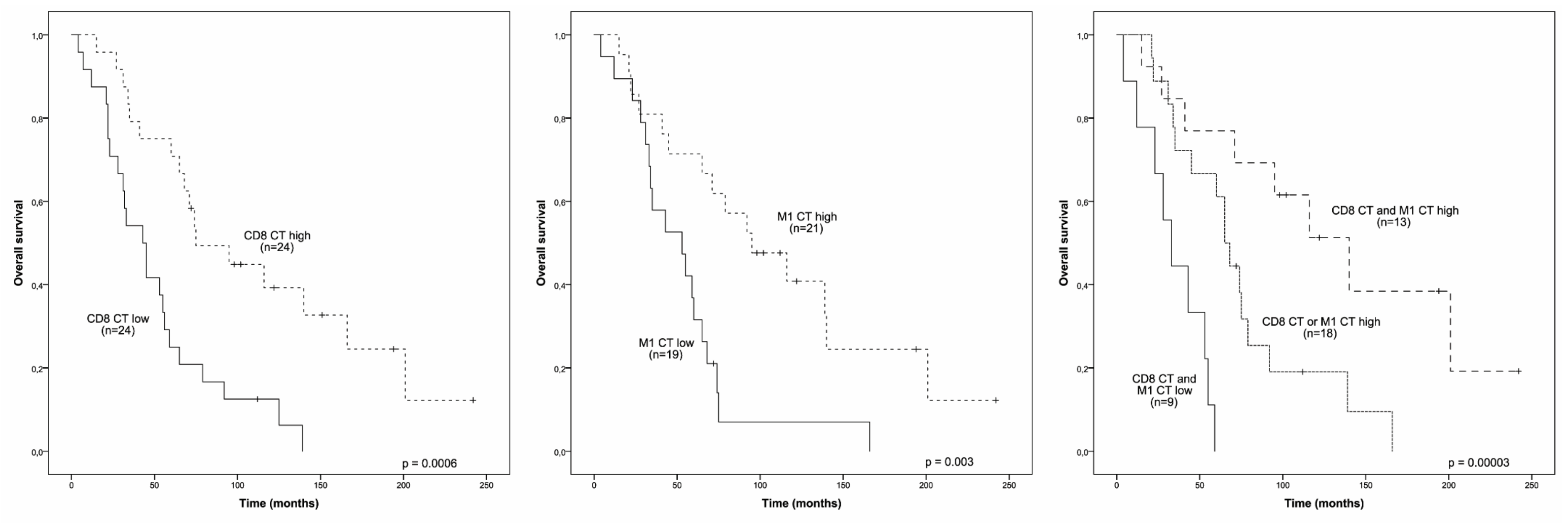

Supplementary figure 1 Survival analysis of $HER2^+$ breast cancer patients in the presence of low and high infiltration of CD8+ T-cells and M1-like macrophages. Survival estimates from Kaplan-Meier analysis, illustrating the difference between the low and high infiltration of $CD8^+$ T-cells and M1 like macrophages in the centre of the tumour separately and together.
